# Supplementary figures and images for: Phosphorylation-Dependent Differences in CXCR4-LASP1-AKT1 Interaction between Breast Cancer and Chronic Myeloid Leukemia
Source: Cells. 2020 Feb 14;9(2):444. doi: 10.3390/cells9020444 (PMC7072741; doi:10.3390/cells9020444)

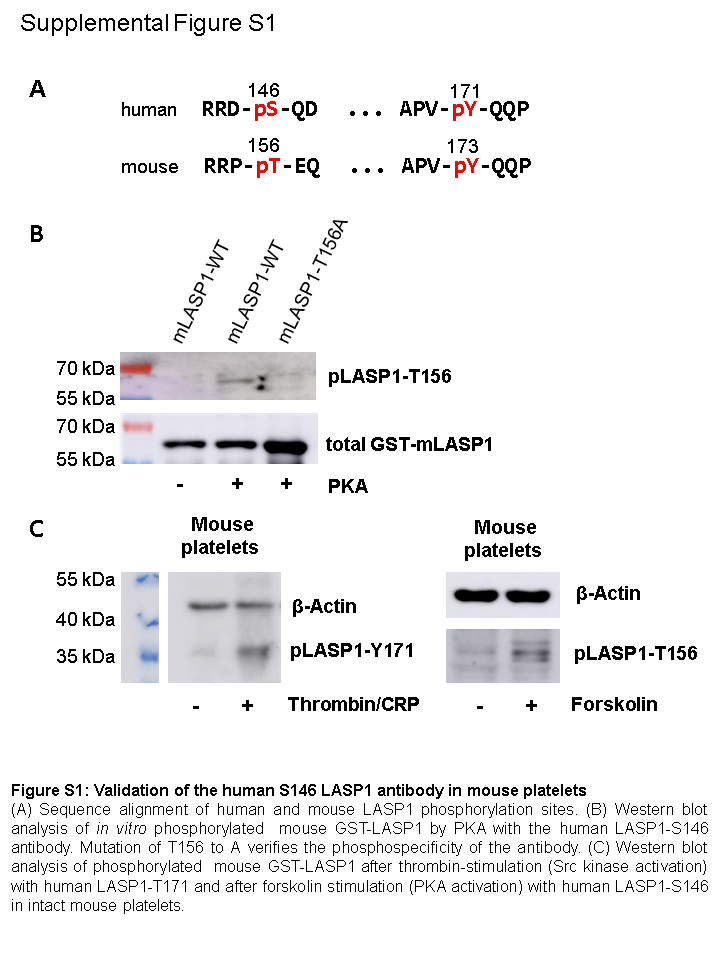

Supplement: Supplementary file 1 [file cells-09-00444-s001.zip › supplementary Materials/Revision Manuscript Supplemental Fig. 1.tif]
